# Magnetic nanoparticles coated with carboxylate-terminated carbosilane dendrons as a reusable and green approach to extract/purify proteins

**DOI:** 10.1007/s00216-021-03794-7

**Published:** 2021-12-09

**Authors:** Isabel M. Prados, Andrea Barrios-Gumiel, Francisco J. de la Mata, M. Luisa Marina, M. Concepción García

**Affiliations:** 1 Departamento de Química Analítica, Química Física e Ingeniería Química, Universidad de Alcalá, Ctra. Madrid-Barcelona Km. 33.600, 28871 Alcalá de Henares, Madrid Spain; 2 Departamento Química Orgánica y Química Inorgánica, Universidad de Alcalá, Ctra. Madrid-Barcelona Km. 33.600, 28871 Alcalá de Henares, Madrid Spain; 3 Instituto de Investigación Química “Andrés M. del Rio” (IQAR), Universidad de Alcalá, Ctra. Madrid-Barcelona Km. 33.600, 28871 Alcalá de Henares, Madrid Spain; 4 Networking Research Center on Bioengineering, Biomaterials and Nanomedicine (CIBER-BBN), Madrid, Spain

**Keywords:** Carbosilane dendron magnetic nanoparticles, Protein, Sustainability, Purification, Extraction

## Abstract

Extraction/purification of proteins, at both analytical and industrial levels, is a limiting step that usually requires the use of organic solvents and involves tedious work and a high cost. This work proposes a more sustainable alternative based on the use of magnetic nanoparticles (MNPs) coated with carboxylate-terminated carbosilane dendrons. MNPs coated with first- and second-generation carbosilane dendrons and bare MNPs were employed for the extraction of proteins with different molecular weights and charges. Interaction of proteins with MNPs significantly varied with the pH, the protein, and the dendron generation (different sizes and number of charges in the periphery). Optimal dendron:protein molar ratios and suitable conditions for disrupting interactions after protein extraction were also researched. Second-generation dendron-coated MNPs showed 100% retention capability for all proteins when using acidic conditions. They were reused without losing magnetism or interaction capacity after a disruption of protein-dendron interactions with 0.2% SDS at 100 °C for 10 min. The capacity of dendron-coated MNPs was successfully applied to the recovery/purification of proteins from two food by-products, olive seeds and cheese whey.

**Graphical abstract:**

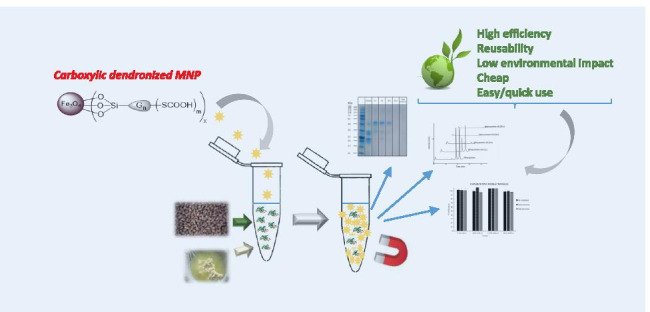

**Supplementary Information:**

The online version contains supplementary material available at 10.1007/s00216-021-03794-7.

## Introduction

Purification and extraction of proteins are a required step in the (bio)analysis of proteins and in the recovery of proteins from food samples or by-products. At the (bio)analytical level, extraction/purification of proteins is a tedious and non-sustainable stage that usually involves the consumption of high amounts of solvents [1]. Indeed, the purification of proteins is mainly carried out by precipitation with organic solvents such as cold acetone and trichloroacetic acid [2, 3]. The separation of a specific proteins is usually carried out by affinity chromatography. At the industrial level, recovery of proteins from food by-products, as example, is usually carried out by membrane filtration and spray-drying, which results in low purification levels and recoveries.

Magnetic nanoparticles (MNPs), mainly based on magnetite (Fe_3_O_4_), have unique properties such as small size, high surface area, easy functionalization, and, above all, paramagnetic properties and easy separation under external magnetic fields [4]. That property makes them to be suitable for separation and purification processes [5]. However, MNPs also present undesirable characteristics such as their intrinsic instability over long periods, their tendency to form agglomerates, their easy oxidation in air resulting in loss of magnetism, and their dispersibility. In order to avoid these limitations, MNPs have been coated with metals [6–8], polymers [9], citrate [10], and bioaffinity ligands [11–13]. MNPs coated with affinity ligands have been employed in the purification of specific proteins such as alkaline phosphatase and α-amylase [14], protein C [15], lysozyme, superoxide dismutase, and lactoferrin [4, 16]. MNPs have also been employed in the purification of proteins from cheese whey [17]. Despite these works demonstrating the capability of MNPs, not one has evaluated their reusability in real matrices, a key fact to raise them as sustainable alternatives to conventional purification/extraction methods. Moreover, the evaluation of the capacity of MNPs to interact with proteins has been studied just with model proteins and not in a real matrix.

Dendrimers are globular-shaped molecules with branches called dendrons that are organized in layers, known as generations. The main feature of these nanoparticles is their multivalent surface [18]. Dendritic nanosystems have demonstrated ability to interact with biomolecules and have been extensively applied in diagnosis, drug delivery, biosensors, catalysis, or even as therapeutic agents and biomimetics. Different types of dendrimers have been developed such as poly(amidoamine) (PAMAM), phosphorus dendrimers, and carbosilane dendrimers, among others. Main differences within them are in their composition and terminal group, which affect their hydrophilic/hydrophobic character and to the kind of interactions they can establish with molecules. Carbosilane dendrimers present a silicon-carbon skeleton and show high thermodynamic, kinetic, and hydrolytic stability. Furthermore, low generations have shown to be biocompatible and soluble in aqueous solutions, when they are properly functionalized with polar groups. They show improved characteristics regarding other dendrimers such as lower toxicity and lipophilic skeleton. Indeed, they present lower toxicity than commercial PAMAM [19], since in this case the use of small generation dendrimers is enough to get the desired biological effect. On the other hand, their lipophilic skeleton facilitates their interaction with biological molecules and membranes.

Our research team has demonstrated the interaction of proteins with carbosilane dendrimers with different functionalities [20–22]. Special attention was paid to carboxylate-terminated carbosilane dendrimers due to their potential to extract and purify proteins. Indeed, González-García et al. [22] confirmed the interaction between proteins and carboxylate-terminated carbosilane dendrimers by computer modeling and applied them to the purification of proteins. Moreover, the research group also studied the interaction of carboxylate-terminated carbosilane dendrons supported on gold nanoparticles with proteins and applied them to the purification of proteins from a complex sample [23]. Despite these promising results, none of these nanomaterials wase easily reutilized, which limited their application in the sustainable extraction/purification of proteins. Indeed, the reusing of these materials involves two steps: the disruption of protein-dendrimer interactions and the separation of dendrimers from proteins. Disruption of interactions sometimes requires highly harsh conditions. By the other hand, the separation of dendrimers from proteins, after interaction disruption, is a difficult target due to the similar size and characteristics of dendrimers and proteins.

This work proposes, for the first time, the use of carboxylate-terminated carbosilane dendrons supported on MNPs (MNPs@G_n_(SCOOH)_m_) as a quick, easy, and sustainable strategy for the extraction/purification of proteins from real matrices. Their magnetic core will make it easier to remove the nanoconjugate from the solution by applying a magnetic field. The high number of carboxylic moieties on the surface of the MNP will favor the interaction with positive domains of proteins and will increase the stability of interactions.

## Materials and methods

### Chemicals and samples

All reagents were of analytical grade. Water was obtained with a Milli-Q system from Millipore (Bedford, MA, USA). Acetone, hexane, hydrochloric acid (HCl), acetic acid (AA), acetonitrile (ACN), and methanol (MeOH) were from Scharlau (Barcelona, Spain). Tris(hydroxymethyl)aminomethane (Tris), sodium dodecyl sulfate (SDS), DL-dithiothreitol (DTT), sodium chloride (NaCl), urea, sodium hydroxide (NaOH), trifluoroacetic acid (TFA), β-mercaptoethanol (β-M), bovine serum albumin (BSA), lysozyme from chicken eggs (LYS), myoglobin from equine heart (MYO), and concanavalin (CONC) were from Merck (Darmstadt, Germany). Laemmli buffer, Mini-Protean precast gels, Tris/glycine/SDS running buffer, Bio-Safe Coomassie stain, and Bradford reagent (Coomassie Blue G-250) were acquired at Bio-Rad (Hercules, CA, USA).

Raw olives of Picual variety were kindly donated by the World Olive Germplasm Bank of IFAPA (Córdoba, Spain) and cheese whey was kindly donated by a cheese factory.

### Magnetic nanoparticles (MNPs)

MNPs coated with first (MNPs@G_1_(SCOOH)_2_) or second (MNPs@G_2_(SCOOH)_4_) generation carbosilane dendrons functionalized with carboxylate groups were synthesized according to a method previously described by Barrios-Gumiel et al. [24]. Briefly, initial dendrons with an amine and an allyl group were employed. The amine group allowed obtaining an alcoxysilane group at the focal point by its reaction with isocyanates. This alcoxysilane group was necessary to anchor the dendron to the MNP. The allyl group allowed introduction of carboxyl groups by reaction with 3-mercaptopropionic acid under UV irradiation through a thiol-ene addition [24]. A narrow size distribution was obtained for both dendron generations. Indeed, particle sizes were 14 ± 3 nm, for the first generation, and 12 ± 2 nm, for the second generation. Additional characterization of materials was reported in reference [24].

**Fig. 1:**
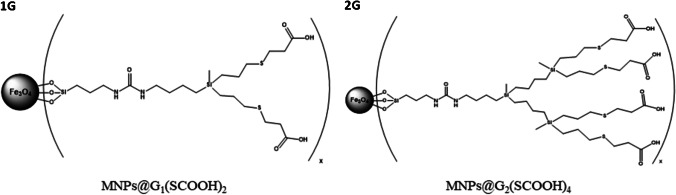
Chemical structure of MNPs@G_1_(SCOOH)_2_ and MNPs@G_2_(SCOOH)_4_

The structures of the first and second generations of these dendrons are shown in Fig. 1. Moreover, bared MNPs, synthesized following the method described by Barrios-Gumiel et al. [24], were also employed to study their interaction with proteins.

### Study of interactions between standard proteins and coated and bared MNPs

Dendron-coated MNPs and bared MNPs were firstly dispersed in water and, next, the pH was adjusted at three different levels (pH = 1.8 (0.2% TFA), pH = 7.0 (H_2_O), and pH = 9.0 (0.1 M Tris–HCl)). Standard proteins were added to these dispersions at different concentrations and mixtures were mixed in a Vortex for 0.5–2 min. A neodymium magnet was next employed for the separation of MNPs from the solution. For that purpose, the magnet was kept over the solution for 20 min. The MNPs were rinsed with Milli-Q water (1 mL) twice and then different solvents and conditions were employed to disrupt interactions established between proteins and MNPs. Blanks obtained under the same conditions, in the absence of proteins, were also obtained. MNPs were washed and stored until next use.

### Transmission electron microscopy (TEM)

Solutions of 1 mg/mL of bared MNPs, MNPs@G_1_(SCOOH)_2_, and MNPs@G_2_(SCOOH)_4_, with and without LYS were sonicated for 20 min in distilled water. A total of 50 µL of these solutions was placed on a copper grid with a carbon surface and dried at 60 °C for 48 h. Transmission electron microscopy images were obtained from a Zeiss EM-10 transmission electron microscope (Oberkochen, Germany).

### Extraction of proteins from olive seeds

Protein extraction was carried out according to the method described by Esteve et al. [25]. Milled and defatted olive seeds (0.03 g) were treated with 5 mL of an extracting buffer (100 mM Tris–HCl, 0.5% SDS, and 0.5% DTT, pH 7.5) using a high-intensity focused ultrasound (HIFU) probe (model VCX130, Sonic Vibra-Cell, Hartford, CT, USA) at 30% of amplitude for 5 min. After centrifugation at 6000* g* for 10 min, the proteins in the supernatant were collected. Extracted proteins were purified by precipitation with cold acetone at a 1:2 protein:acetone ratio.

### Purification/extraction of proteins from real samples using MNPs@G_2_(SCOOH)_4_

MNPs@G_2_(SCOOH)_4_ were employed for the purification of proteins extracted from olive seeds and proteins present in cheese whey. MNPs@G_2_(SCOOH)_4_ were dispersed in water and different concentrations of proteins (from olive seeds or whey) were added. Afterwards, the pH was adjusted to 1.8 using a TFA solution to promote protein interactions with MNPs and, then, the mixture was kept over a neodymium magnet for 20 min. The supernatant was next separated from the MNPs@G_2_(SCOOH)_4_ holding the proteins. Proteins remaining in solution were next determined to evaluate the percentage of retained proteins. After rinsing MNPs@G_2_(SCOOH)_4_ twice with water, optimum solvent and conditions were employed to disrupt interactions and release purified/extracted proteins.

### Separation and determination of proteins

Due to the fluorescence and UV–Vis absorption of dendron-coated nanoparticles, the amount of proteins interacting with MNPs was evaluated from the amount of proteins in the initial solution and those remaining in solution after interacting with MNPs. The content of proteins in solution was determined by UV–Vis spectroscopy using Bradford assay. In those cases where it was not possible to use this assay due to the interferences of SDS, proteins in solution were visualized by SDS-PAGE.

#### Bradford assay

Protein content in solution was estimated following the Bradford method [26]. Bradford reagent was diluted five times to prepare the Bradford solution. A volume of 1 mL was added to 12.3 µL of sample and the absorbance of the mixture was measured at 595 nm after 5 min in a UV/Vis Agilent Cary 8454 spectrophotometer from Agilent Technologies (Palo Alto, CA, USA). The percentage of retained proteins was calculated according the following equation:

$$\mathrm{\%\:retained\:protein}= \frac{{\mathrm{C}}_{\mathrm{i}}-{\mathrm{C}}_{\mathrm{s}}}{{\mathrm{C}}_{\mathrm{i}}} \times 100$$

where *C*_i_ is the concentration of added proteins and *C*_s_ the concentration of proteins in the supernatant after mixing with MNPs@G_n_(SCOOH)_m_.

#### Sodium dodecyl sulfate–polyacrylamide gel electrophoresis (SDS-PAGE)

A Bio-Rad Mini-protean system (Hercules, CA, USA) was used for the SDS-PAGE separation of proteins. In total, 15 µL of Laemmli buffer containing 5% of β-mercaptoethanol was mixed with 15 µL of protein/sample and the mixture was heated at 100 °C for 10 min. The mixtures were loaded into commercial-ready precast gels. The protein separation was carried out by applying 75 V for 5 min and 150 V until separation completion using Tris/glycine/SDS as running buffer. Molecular markers of standard proteins with molecular weights from 10 to 250 kDa were also run. After separation, proteins were fixed using 50 mL of a mixture of acetic acid/methanol/water (10:40:50) by shaking for 30 min. After mixture removal, the gel was stained with 50 mL of Bio-Safe Coomassie stain for 1 h, rinsed with water for 1 h to remove remaining buffer, and scanned with an Epson perfection V39 scanner (Suwa, Japan).

#### Separation of proteins by RP-HPLC

Cheese whey proteins were separated by RP-HPLC using a HPLC equipment from Agilent Technologies (Pittsburgh, PA, USA) model 1100, equipped with a vacuum degasser, a quaternary pump, an automatic injection system, a thermostatic column compartment, a diode array detector, and a fluorescence detector. Control of the equipment and data acquisition were performed with the HP ChemStation software. The separation was carried out in an Aeris Widepore XB-C18 column (250 × 4.6 mm, 3.6 µm particle size) with an Aeris Widepore XB-C18 guard column, both from Phenomenex (Torrance, CA, USA). The separation of cheese whey proteins was carried out following the method described by García et al. [27] with slight modifications. The mobile phases consisted of water with 0.1% TFA (v/v) (phase A) and ACN with 0.1% TFA (v/v) (phase B). The flow rate was 0.3 mL/min and the injection volume was 15 µL. The elution gradient was as follows: 20% B for 1 min, 20–42% B in 15 min, 42–46% B in 4 min, 46–100% B in 0.5 min, 100% B for 0.5 min, and finally from 100 to 20% B in 0.5 min.

### Statistical analysis

Statistical analysis was performed using Statgraphics Software Plus 5.1 (Statpoint Technologies, Inc., Warranton, VA, USA). Data comparison was carried out by one-way analysis of variance (ANOVA). Fisher’s Multiple Range test was used to determine statistically significant differences (*p*-value < 0.05) between mean values from different samples at 95% confidence level. All data were expressed as mean ± standard deviation of three measurements of two independent experiments.

## Results and discussion

New trends in the extraction/purification of proteins are focused on sustainable and reusable proposals. Carboxylate-terminated carbosilane dendrimers supported on gold nanoparticles have been proposed to extract/purify proteins from complex samples [23]. However, the application of these nanomaterials is frequently limited by the difficult disruption of the established interactions or by the tricky separation of nanoparticles from proteins after disruption of interactions. This work evaluates the potential of carboxylate-terminated carbosilane dendrons supported on MNPs for the sustainable sample preparation of proteins.

### Evaluation of the interaction of MNPs@G_n_(SCOOH)_m_ and bared MNPs with proteins

MNPs coated with first- and second-generation carboxylate dendrons (MNPs@G_n_(SCOOH)_m_) were employed in this study (see Fig. 1). Four standard proteins (LYS, MYO, CONC, and BSA) with different molecular weights and isoelectric points (see Table 1) were treated with different concentrations of MNPs@G_n_(SCOOH)_m_ to demonstrate their interaction. Experiments were carried out at three different pHs (1.8, 7.5, and 9.0). All proteins showed positive charge at acid pH while, at higher pH, LYS presented a positive charge and BSA, CONC, and MYO presented negative charge. Conversely, carboxylate groups in dendrons were not charged at pH = 1.8, whereas they were negatively charged at higher pHs.

**Table 1:** Molecular weights and isoelectric points of standard proteins employed in this work

Protein	LYS	MYO	CONC	BSA
Isoelectric point	11.35	6.80	4.50–5.50	4.70
Molecular weight (kDa)	14.3	17.8	25.5	66.5

**Fig. 2:**
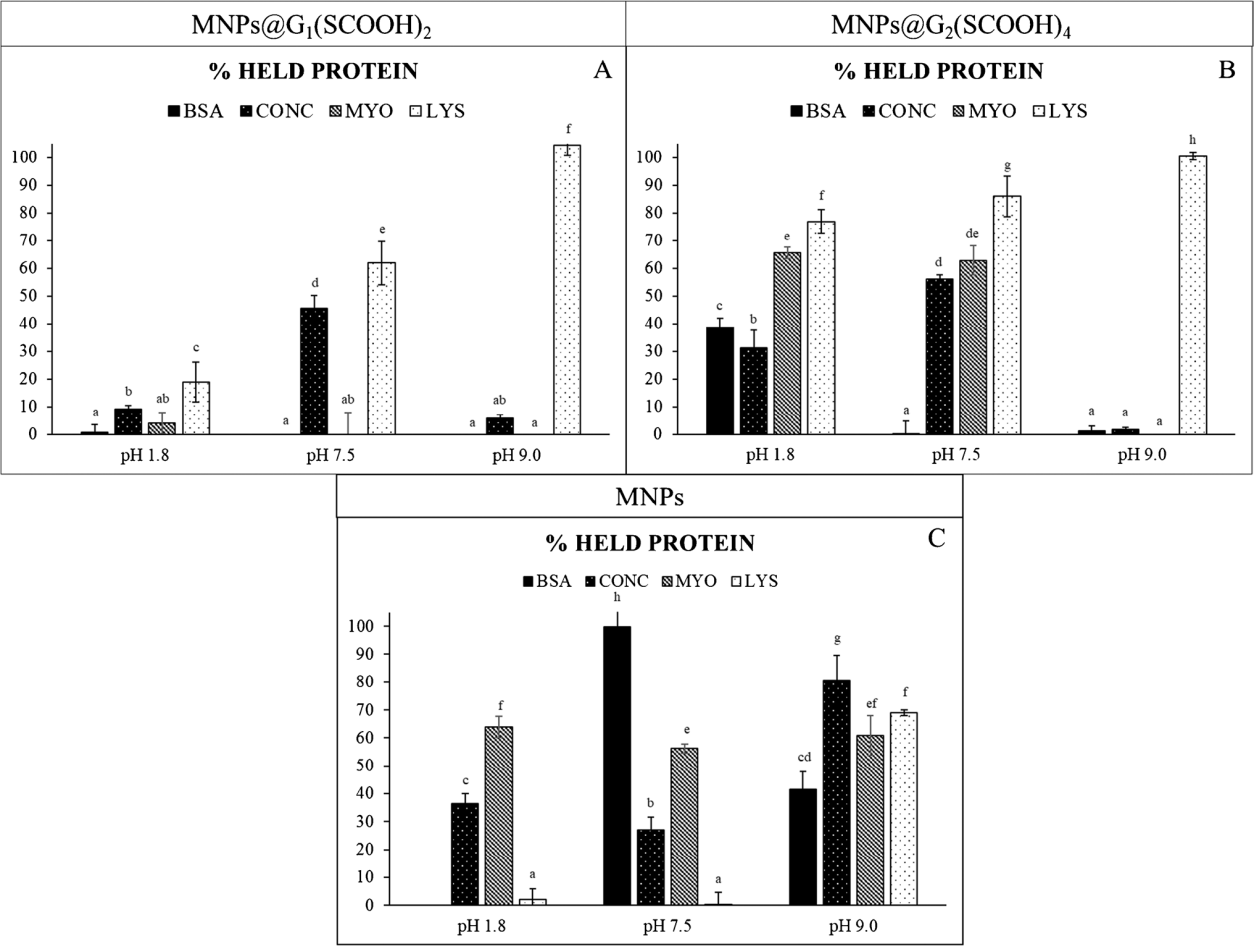
Percentage of proteins (BSA, CONC, MYO, and LYS) retained on MNPs@G_1_(SCOOH)_2_ (A), MNPs@G_2_(SCOOH)_4_ (B), and bared MNPs (C) under different pH conditions (1.8, 7.5, and 9.0)

Different dendron:protein molar ratios were firstly employed (1:1, 4:1, 20:1, 50:1, 100:1, and 500:1) to evaluate the interaction of all proteins with MNPs. A dendron:protein ratio of 100:1 was selected for all proteins (LYS, MYO, and CONC) with the exception of BSA that required a higher ratio (500:1) for the establishment of interactions with MNPs. Under these dendron:protein ratios, the percentage of proteins retained on MNPs was calculated as the difference between the concentration of proteins in solution before and after interaction with MNPs. Figure 2A and B show the percentage for every protein at different pHs for MNPs@G_1_(SCOOH)_2_ and MNPs@G_2_(SCOOH)_4_. The highest interaction of dendron-coated MNPs and proteins, in general, was observed with the MNPs@G_2_(SCOOH)_4_, probably due to the highest number of functional groups. Similar results were observed when using dendron-coated gold nanoparticles and carboxylate-terminated carbosilane dendrimers [20, 21]. Interaction with MNPs@G_1_(SCOOH)_2_ was only significant for LYS at basic pH.

The interaction of proteins with MNPs@G_2_(SCOOH)_4_ greatly varied with the pH. All proteins interacted with MNPs@G_2_(SCOOH)_4_, at some extent, at acid pH observing the highest interaction in the case of LYS. At this pH, all proteins were positively charged while dendrons were uncharged. On the other hand, BSA, CONC, and MYO showed no interaction at basic pH with MNPs@G_2_(SCOOH)_4_ while LYS was the only one that could interact at this pH. This behavior can be explained taking into account that LYS, under basic conditions (pH = 9.0), is positively charged while the rest of proteins showed negative charges as dendrons. Taking into account these results, electrostatic forces seem to be the main contributors to interactions at basic pH while hydrophobic interactions could be occurring at lower pHs.

Since bared MNPs have demonstrated to be effective adsorbents for proteins and other biomolecules [5], results obtained using MNPs@G_2_(SCOOH)_4_ were compared with those observed with bared MNPs (Fe_3_O_4_ nanoparticles) to evaluate the contribution of dendrons (results included in Fig. 2C). Interaction of proteins with bared MNPs was very different to that observed with coated MNPs. At acid pH, interaction of BSA and LYS with MNPs@G_2_(SCOOH)_4_ was higher than that with bared MNPs while interaction of CONC and MYO with bared MNPs was similar to that observed for MNPs@G_2_(SCOOH)_4_. At neutral pH, BSA showed no retention on MNPs@G_2_(SCOOH)_4_ while it was highly retained on bared MNPs. Moreover, LYS and CONC showed a higher retention on MNPs@G_2_(SCOOH)_4_, at this pH, while a similar retention on both bared MNPs and MNPs@G_2_(SCOOH)_4_ was observed for MYO. Main differences were observed at pH = 9.0, when all proteins were negatively charged except LYS. At this pH, proteins were more retained on bared MNPs than in MNPs@G_2_(SCOOH)_4_, with the exception of LYS. Since bared MNPs are uncharged at all pHs, chemical forces involved in the interactions with proteins were not electrostatic.

**Fig. 3:**
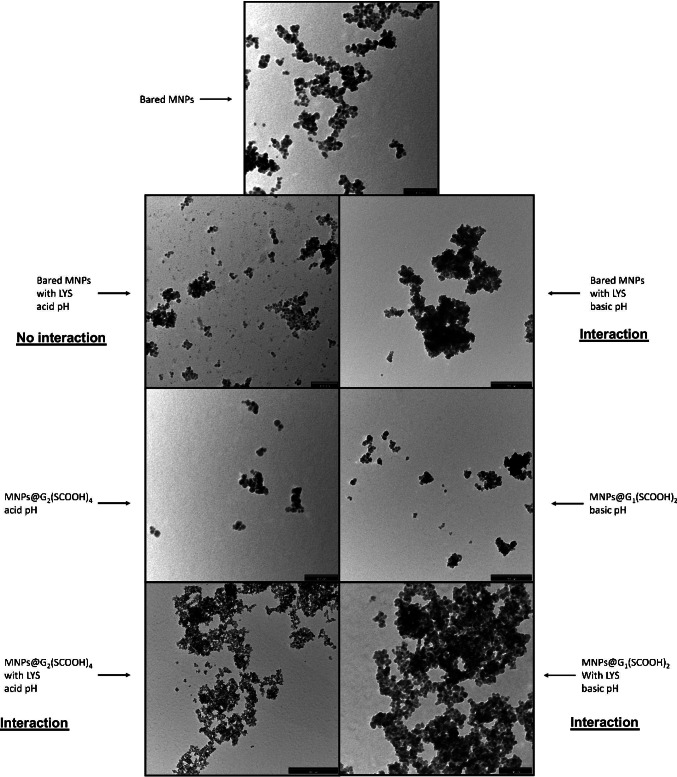
Transmission electron microscopy (TEM) images of bared MNPs, MNPs@G_1_(SCOOH)_2_, and MNPs@G_2_(SCOOH)_4_, alone and with LYS at different pHs

Figure 3 shows TEM images corresponding to bared MNPs alone and in the presence of LYS (at acidic pH (no interaction) and at basic pH (interaction with proteins); MNPs@G_1_(SCOOH)_2_ alone and in the presence of LYS at basic pH (interaction with proteins); and MNPs@G_2_(SCOOH)_4_ with and without LYS at acid pH (interaction with proteins). The main difference observed between the image corresponding to bared MNPs and that with LYS as acid pH, when no interaction was observed (see Fig. 2), was the presence of small spots in the solution that could correspond to LYS. These spots did not appear in the other images in the presence of LYS, probably because they were taken under conditions promoting the interaction with MNPs. Moreover, images also showed that the tendency of nanoparticles to form aggregates was higher when they were interacting with LYS (images corresponding to bared MNPS with LYS at basic pH, MNPs@G_1_(SCOOH)_2_ with LYS at basic pH, and MNPs@G_2_(SCOOH)_4_ with LYS at acid pH) than when they were alone.

### Study of conditions promoting the interaction between proteins and MNPs and those enabling the disruption of established interactions. Evaluation of the application of MNPs in successive extraction/purifications

Further investigations were carried out in order to find the best conditions for the establishment and disruption of interactions with the different MNPs. These conditions were next employed in the study of the reusability of MNPs.

#### Interaction of proteins with MNPs@G_1_(SCOOH)_2_

The only significant interaction of proteins with MNPs@G_1_(SCOOH)_2_ was observed at pH 9.0 for LYS (see Fig. 2). Under this pH, different dendron:protein molar ratios (from 12.5:1 to 100:1) were employed to find out the most suitable conditions to hold 100% of proteins. Results in Table 2 demonstrated that the highest retention of LYS was observed at dendron:protein ratios higher than 75:1. Moreover, this interaction was easily disrupted by reducing the pH with 0.2% TFA. Table 2 also shows that there were no significant differences between the percentage of retained LYS and eluted proteins at every dendron:protein ratio, which confirmed that 0.2% TFA was suitable for disrupting the interaction. Furthermore, the effect of the variation of the interaction time on the retention of LYS was also evaluated (using times from 0.5 to 2.0 min), not observing significant differences in the percentage of retained protein.

**Table 2:** Percentage of retained LYS at different dendron:protein ratios using MNPs@G_1_(SCOOH)_2_ at pH = 9 and percentage of released LYS (related to the initial LYS)

Dendron:protein molar ratio	100:1	75:1	50:1	25:1	12.5:1
% retained proteins	106 ± 5^a^	98 ± 6^b^	76 ± 1^c^	50 ± 4^d^	33 ± 3^e^
% eluted proteins (0.2% TFA)	107 ± 6^a^	103 ± 4^b^	73 ± 3^c^	48 ± 2^d^	27 ± 3^e^

^a^^, b, c, d, e^ Different superscript letters denote values that are statistically different (*p*-value < 0.05)

The next step was the evaluation of the capability of MNPs@G_1_(SCOOH)_2_ in successive extractions. Nanoparticles were rinsed twice with Milli-Q water between extractions. Electronic Supplementary Material Figure S1 shows the percentage of retained and eluted LYS in three consecutive extractions carried out at the different dendron:protein ratios. No statistical differences between retained and eluted proteins within the same dendron:protein ratio were observed (*p*-value > 0.05). The results demonstrated that the MNPs@G_1_(SCOOH)_2_ could be reused and proposed as a sustainable alternative to other methods employed for the extraction/purification of proteins with high isoelectric points such as LYS.

#### Interaction of proteins with MNPs@G_2_(SCOOH)_4_

**Fig. 4:**
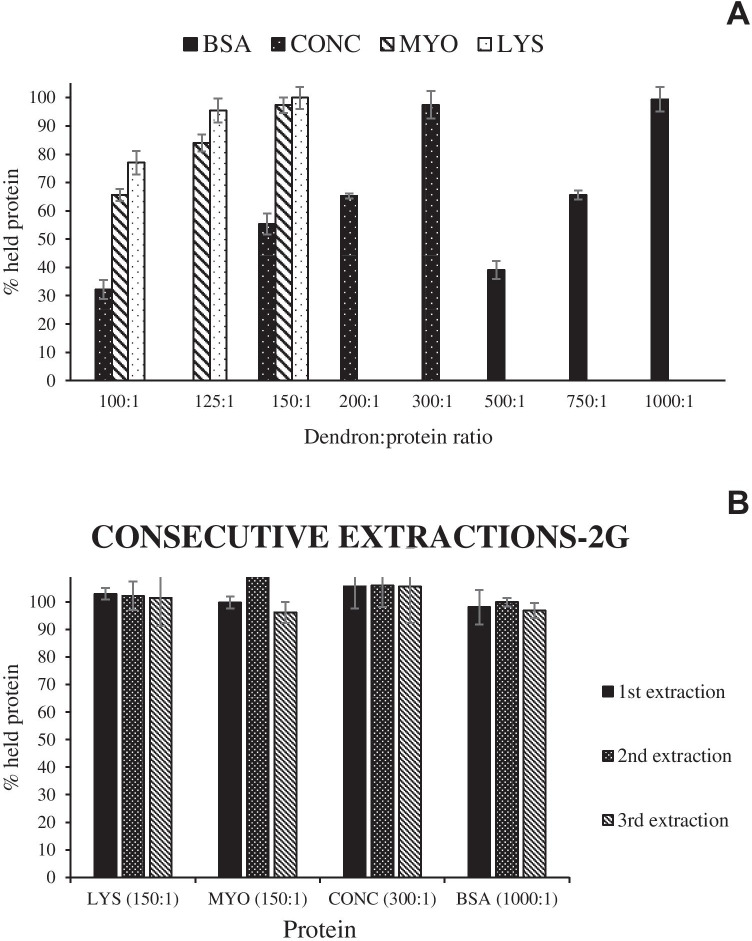
(A) Percentage of proteins (LYS, MYO, CONC, and BSA) retained on MNPs@G_2_(SCOOH)_4_ using different dendron:protein ratios. (B) Percentage of proteins retained on MNPs@G_2_(SCOOH)_4_ in three consecutive extractions, under optimized conditions

All employed standard proteins interacted, in some extent, with MNPs@G_2_(SCOOH)_4_ at acid pH._._ Under these conditions, the effect of the dendron:protein ratio on the retention of every protein was studied and the results are shown in Fig. 4A. Again, the retention of proteins was significantly affected by the dendron:protein ratio (different ratios were tried to reach 100% retention for every protein) (*p*-value < 0.05). While MYO and LYS showed a 100% retention at a 150:1 molar ratio, a higher ratio was required to retain bigger proteins such as CONC (300:1) and BSA (1000:1). This behavior is clearly related with the molecular weight of proteins (see data in Table 1). The higher the molecular weight, the higher the dendron:protein ratio required for the full retention of proteins. Furthermore, the effect of the time on the retention of proteins was also studied but, again, it did not affect the retention of proteins.

**Fig. 5:**
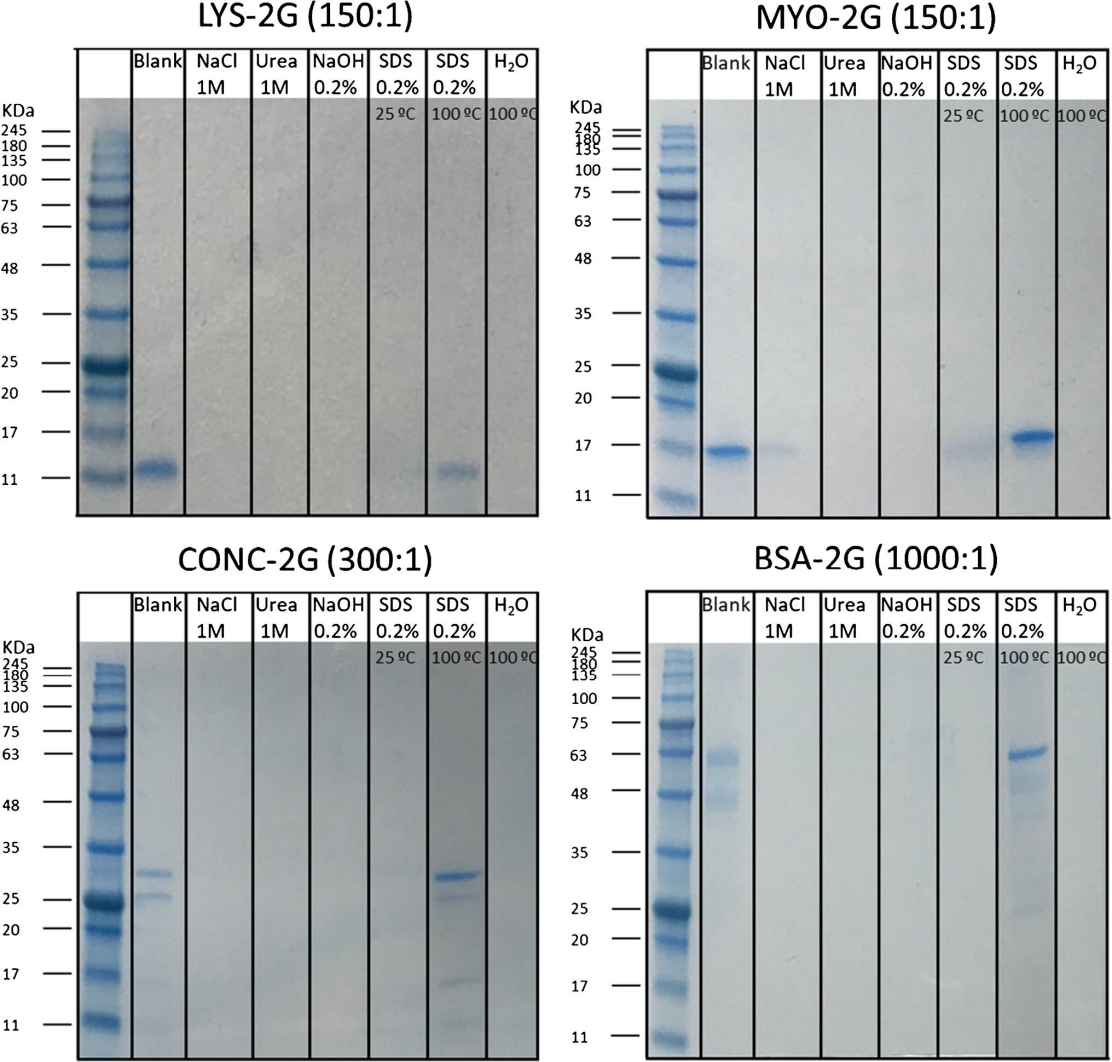
SDS-PAGE profiles obtained with solutions containing LYS, MYO, CONC, and BSA (blanks) and with solutions containing proteins eluted from MNPs@G_2_(SCOOH)_4_ using 1 M NaCl, 1 M urea, 0.2% NaOH, 0.2% SDS at room temperature and at 100 °C, and water at 100 °C

Using these interacting conditions, different media and temperatures were employed to disrupt the interactions established between proteins and MNPs@G_2_(SCOOH)_4_: urea 1 M, NaCl 1 M, NaOH 0.1 M, 0.2% TFA, 0.2% SDS at room temperature and at 100 °C, and water at 100 °C. Figure 5 shows the SDS-PAGE separation of proteins eluting under previous conditions. NaCl is the usual medium employed for the disruption of electrostatic interactions. The lack of disruption confirmed that proteins were retained on MNPs@G_2_(SCOOH)_4_, at acid pH, by other kinds of interactions. Best conditions for the disruption of interactions for the four proteins were 0.2% of SDS at 100 °C for 10 min. The strongest conditions were also applied (SDS + β-mercaptoethanol and Laemmli buffer) after disrupting proteins-MNPs@G_2_(SCOOH)_4_ interactions with 0.2% of SDS at 100 °C for 10 min. Results demonstrated that all proteins were released with 0.2% SDS at 100 °C for 10 min and not one remained on the nanoparticle.

Using optimized conditions, the possibility to use MNPs@G_2_(SCOOH)_4_ for consecutive experiments was next evaluated. Percentages of proteins retained on nanomaterial in three consecutive extractions are shown in Fig. 4B. MNPs@G_2_(SCOOH)_4_ was successfully used in successive extraction of the four proteins without losing their capability. In fact, no significant differences (*p*-value > 0.05) were observed among proteins retained in successive extraction.

#### Interaction of proteins with bared MNPs

Bared MNPs were employed for the successive extraction of standard proteins using conditions previously optimized for disrupting interactions between proteins and MNPs@G_2_(SCOOH)_4_ (0.2% SDS at 100 °C for 10 min). Nevertheless, and regardless the pH, it was observed a decrease in the retention of proteins after the first extraction (see Figure S2 as an example obtained at acid pH). Moreover, Figure S2 also shows a detail of the color of the solution remaining after the first extraction using bared MNPs in comparison with MNPs@G_2_(SCOOH)_4_, observing a red solution corresponding to iron oxide in the case of the bared MNP. These results demonstrated that non-coated MNPs lose their magnetisms and capacity to retain proteins after the first extraction and that they could not be used in successive experiments at any of the three tested pHs. Furthermore, these results also highlighted the key role of the dendron coating in the sustainable use of MNPs in protein sample preparation.

### Application of MNPs@G_2_(SCOOH)_4_ to the purification/extraction of proteins in real matrices

MNPs@G_2_(SCOOH)_4_ were employed for the purification of proteins extracted from a by-product resulting from the olive industry (the olive seeds) and for the recovery of proteins from a by-product resulting from the dairy industry (the cheese whey).

#### Application of MNPs@G_2_(SCOOH)_4_ to the purification of proteins from an olive seed extract

Protein extraction usually involves the use of buffer solutions or organic solvents. Due to the low selectivity of these procedures, it is quite usual to include a last step for the purification of proteins that generally involves their precipitation with cold acetone. Indeed, precipitation with cold acetone has been demonstrated to be very efficient for the extraction/purification of proteins and has been applied for the detection of adulterations of hazelnut oil proteins in extra virgin olive oil [3], among others. Nevertheless, despite its efficiency, this reagent is not suitable for the food industry nor from the environmental point of view. Moreover, the time required for that precipitation is high; in many occasions, it is overnight. This work proposes a greener alternative to the purification of proteins using cold acetone based on the use of MNPs@G_2_(SCOOH)_4_. This proposal will be applied to the purification of proteins, previously extracted from olive seeds using a method described by Esteve et al. [25]. Olive seed is a by-product from the olive industry with a high protein content that is underused and undervalued. Purification of olive seed proteins, after extraction, has been carried out by precipitation with cold acetone for 1 h [25].

**Fig. 6:**
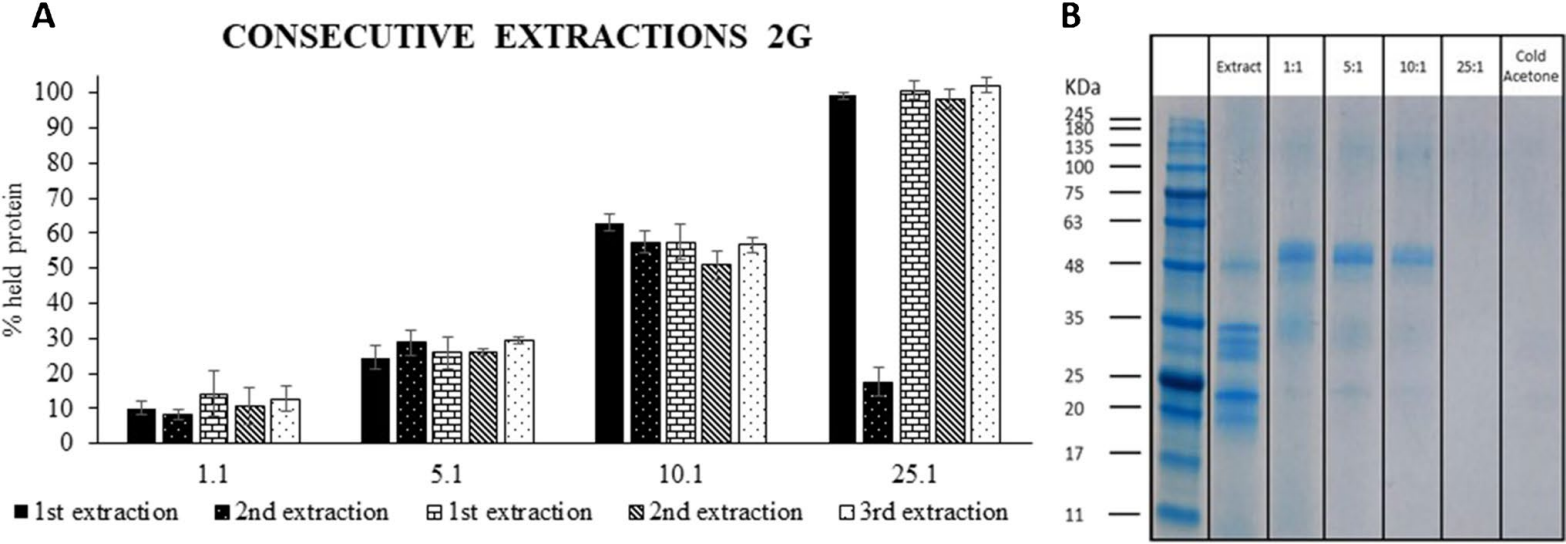
(A) Percentage of olive seed proteins retained on MNPs@G_2_(SCOOH)_4_ in five consecutive experiments using different dendron:protein ratios; (B) SDS-PAGE profiles corresponding to olive seed proteins remaining in solution after treatment with MNPs@G_2_(SCOOH)_4_ at different dendron:protein ratios or after precipitation with cold acetone precipitation

Based on the abovementioned results, purification was carried out at acid pH using dendron:protein ratios from 1:1 to 25:1 (w/w). Figure 6A shows the percentages of retained proteins at the different dendron:protein ratios. The percentage of retained proteins increased with the dendron concentration up to a 25:1 ratio that resulted in a 100% of protein retention (bar in black at Fig. 6A). Moreover, this experiment was carried out five consecutive times to evaluate the reusability of MNPs@G_2_(SCOOH)_4_. The capacity of MNPs@G_2_(SCOOH)_4_ to interact with proteins did not change after five consecutive experiments (*p*-value > 0.05) when using 1:1, 5:1, and 10:1 dendron:protein ratios, employing in all cases 0.2% SDS at 100 °C for 10 min, to disrupt interactions after purification. However, when a 25:1 dendron:protein ratio was used in a second experiment, the retention decreased to 20%. This is because the higher retention of proteins at this dendron:protein ratio made such that the conditions employed to disrupt interactions (0.2% SDS, 100 °C, 10 min) were not strong enough to release proteins. These unreleased proteins block retention sites of the MNPs@G_2_(SCOOH)_4_ resulting in a significant reduction in protein retention in the next experiment. An increase in the percentage of SDS from 0.2 to 0.4% enabled release of all retained proteins and accommodation of the nanomaterial for the next experiment. Therefore, it was possible to reach 100% retention in the third, fourth, and fifth experiments (see Fig. 6B).

These results were compared with those obtained by precipitation with cold acetone [25]. Figure 6B shows the electrophoretic separation of proteins remaining in solution after treatment with different dendron:protein ratios and with cold acetone. Purification obtained using MNPs@G_2_(SCOOH)_4_ at a 25:1 dendron:protein ratio (100% retention) was as efficient as that observed when doing the precipitation with acetone, such as in the work reported by Esteve et al. [25]. Moreover, the proposed methodology enabled the reusability of MNPs@G_2_(SCOOH)_4_ that makes this method more sustainable, with a lower environmental impact, and cheaper than the precipitation of proteins with an organic solvent. Moreover, the use of MNPs@G_2_(SCOOH)_4_ is less time-consuming and tedious than the precipitation with acetone [25].

#### Application of MNPs@G_2_(SCOOH)_4_ to the recovery of proteins from cheese whey

Cheese whey is a by-product from the dairy industry containing proteins (mainly, α-lactoalbumin (α-LA) and β-lactoglobulins (β-LG (A + B))) with high biological and economic value. Recovery of proteins from cheese whey is usually carried out by membrane filtration or spray-drying. A recent alternative has been the use of MNP coated with Cu^2+^ and/or citric acid [17]. Nevertheless, the authors reported just the partial recovery of whey proteins (from 14 to 60% of protein reduction in the whey) and no reusability was possible since part of the proteins remained in the own MNP. This work purposes the application of MNPs@G_2_(SCOOH)_4_ for the recovery of proteins from cheese whey.

**Fig. 7:**
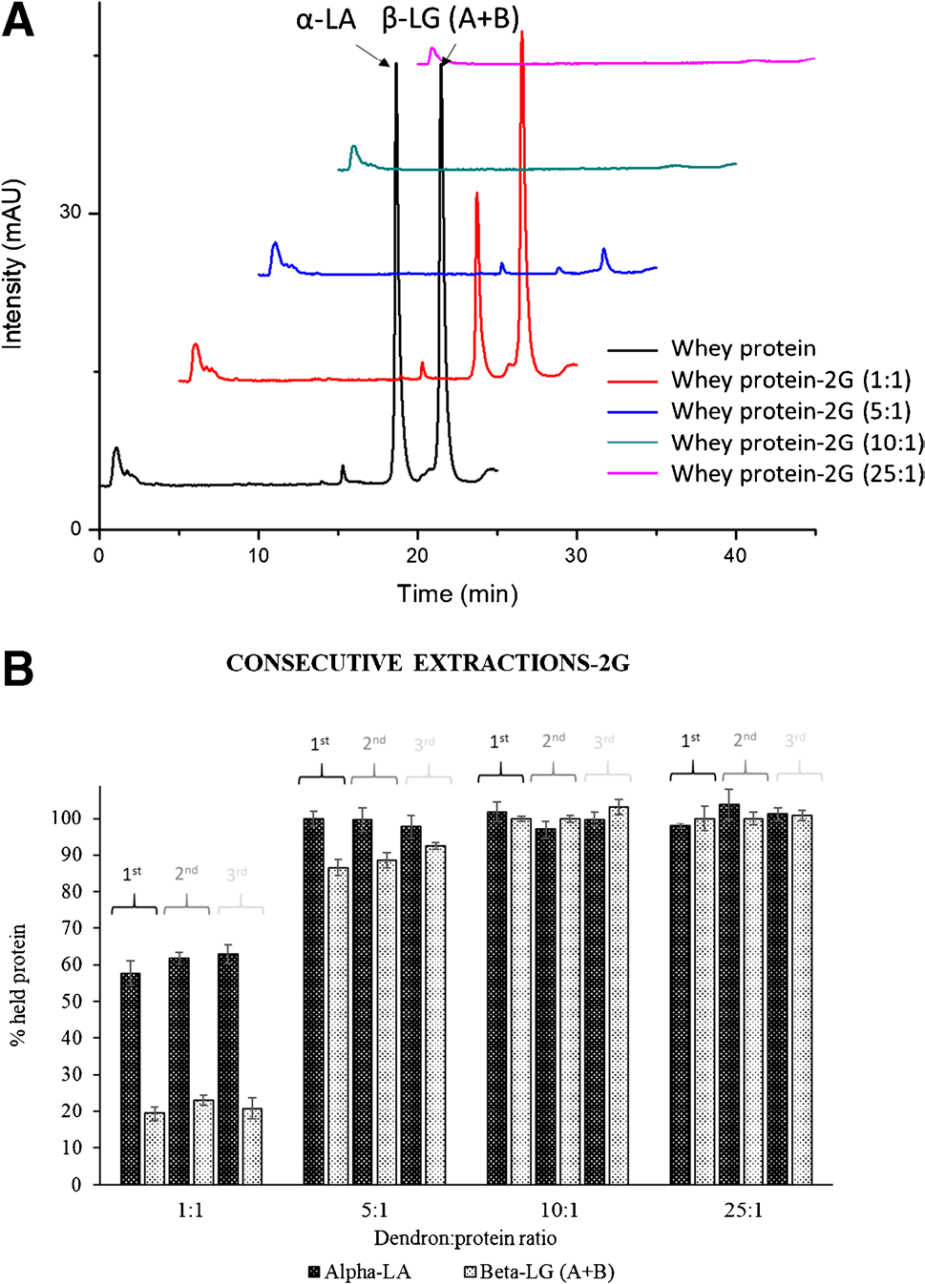
(A) RP-HPLC profile corresponding to cheese whey proteins remaining in solution (non-retained) after treatment with MNPs@G_2_(SCOOH)_4_ at different dendron:protein ratios. (B) Percentage of cheese whey proteins retained on MNPs@G_2_(SCOOH)_4_ in three consecutive experiments using different dendron:protein ratios

Protein extraction was carried out under the same conditions previously employed for the purification of olive seed proteins (acid pH and dendron:protein ratios from 1:1 to 25:1 (w/w)). Figure 7A shows the chromatographic separation of α-LA and β-LG (A + B) remaining in the whey after treatment with different dendron:protein ratios. At a 1:1 dendron:protein ratio, only α-LA was partially retained while almost no retention was observed for β-LG (A + B) that coeluted. Figure 7B showed that the retention of proteins increased with the dendron concentration, obtaining 100% proteins retention at dendron:protein ratios equal or higher than 5:1, for α-LA, and 10:1, for β-LG (A + B). Furthermore, this experiment was carried out three consecutive times, by the disruption of interactions with 0.4% SDS at 100 °C for 10 min. Figure 7B shows the total recovery of whey proteins in successive extraction, not observing statistical differences among retained proteins (*p*-value > 0.05).

Comparing with the work reported by Nicolás et al. [17], the novel method enables the total recovery of whey proteins, since MNPs@G_2_(SCOOH)_4_ can retain all whey proteins and the use of 0.4% SDS at 100 °C for 10 min can elute all absorbed proteins. This contrasts with that reported previously [17]. In fact, MNP coated with Cu^2+^/citric acid never yielded 100% adsorption of whey proteins and, additionally, it was not possible to recover all adsorbed proteins, thus resulting in a partial cleaning of whey. Moreover, these MNPs were not employed for successive extractions. This demonstrates the interest of MNPs@G_2_(SCOOH)_4_ as a sustainable alternative to conventional methodologies and other more novel approaches employed for the recovery of proteins from aqueous residues, such as cheese whey.

## Conclusions

This work shows the capacity of magnetic nanoparticles coated with carboxylate-terminated carbosilane dendrons to interact with proteins and demonstrates that they are a green alternative to conventional methods employed for the purification/extraction of proteins. Bared and dendron-coated MNPs could interact with standard proteins but bared MNPs lose their magnetism and retention capability after first protein extraction. MNPs coated with first-generation dendrons presented a lower capability to interact with proteins than MNPs with second-generation coating. Suitable conditions for the retention of proteins on second-generation–coated MNPs were acid pH and dendron:protein ratios that depended on the protein molecular weight, requiring a lower ratio for smaller proteins. Second-generation coated MNPs could be used several times after disruption of protein-dendron interactions with 0.2–0.4% SDS at 100 °C for 10 min. These MNPs were successfully employed for the purification of proteins from an olive seed extract and for the recovery of proteins from cheese whey, demonstrating they could be reused. Therefore, upon a slight optimization of the protein:dendron ratio and the conditions to disrupt interactions, second-generation dendron-coated MNPs are a solid alternative to conventional methods employed in protein sample preparation. They show high efficiency in retaining proteins and are reusable, environmentally friendly, cheap, and easy/quick to use. Further researches are required for scaling these procedures.

## Supplementary Information

Below is the link to the electronic supplementary material.

Supplementary file1 (DOCX 204 KB)

## Data Availability

All data generated or analyzed during this study are included in this published article.

## Code availability

No applicable.
