# Supplementary material for: Magnetic nanoparticles coated with carboxylate-terminated carbosilane dendrons as a reusable and green approach to extract/purify proteins
Source: Anal Bioanal Chem. 2021 Dec 9;414(4):1677–89. doi: 10.1007/s00216-021-03794-7 (PMC8761721; doi:10.1007/s00216-021-03794-7)
Supplement: Supplementary file 1 — Supplementary file1 (DOCX 204 KB) [file 216_2021_3794_MOESM1_ESM.docx]

**SUPPLEMENTARY MATERIAL**

**Magnetic nanoparticles coated with carboxylate-terminated carbosilane dendrons as a reusable and green approach to extract/purify proteins**

Isabel M. Prados^1^, Andrea Barrios-Gumiel^2^, Francisco J. de la Mata^2,3,4^, M. Luisa Marina^1,3^, M. Concepción García^1,3^*

^1^Departamento de Química Analítica, Química Física e Ingeniería Química, Universidad de Alcalá, Ctra. Madrid-Barcelona Km. 33.600, 28871 Alcalá de Henares (Madrid), Spain.

^2^Departamento Química Orgánica y Química Inorgánica, Universidad de Alcalá, Ctra. Madrid-Barcelona Km. 33.600, 28871 Alcalá de Henares (Madrid), Spain.

^3^Instituto de Investigación Química "Andrés M. del Rio" (IQAR), Universidad de Alcalá, Ctra. Madrid-Barcelona Km. 33.600, 28871 Alcalá de Henares (Madrid), Spain.

^4^ Networking Research Center on Bioengineering, Biomaterials and Nanomedicine (CIBER-BBN)

* Corresponding author

concepcion.garcia@uah.es

P: +34 918854915


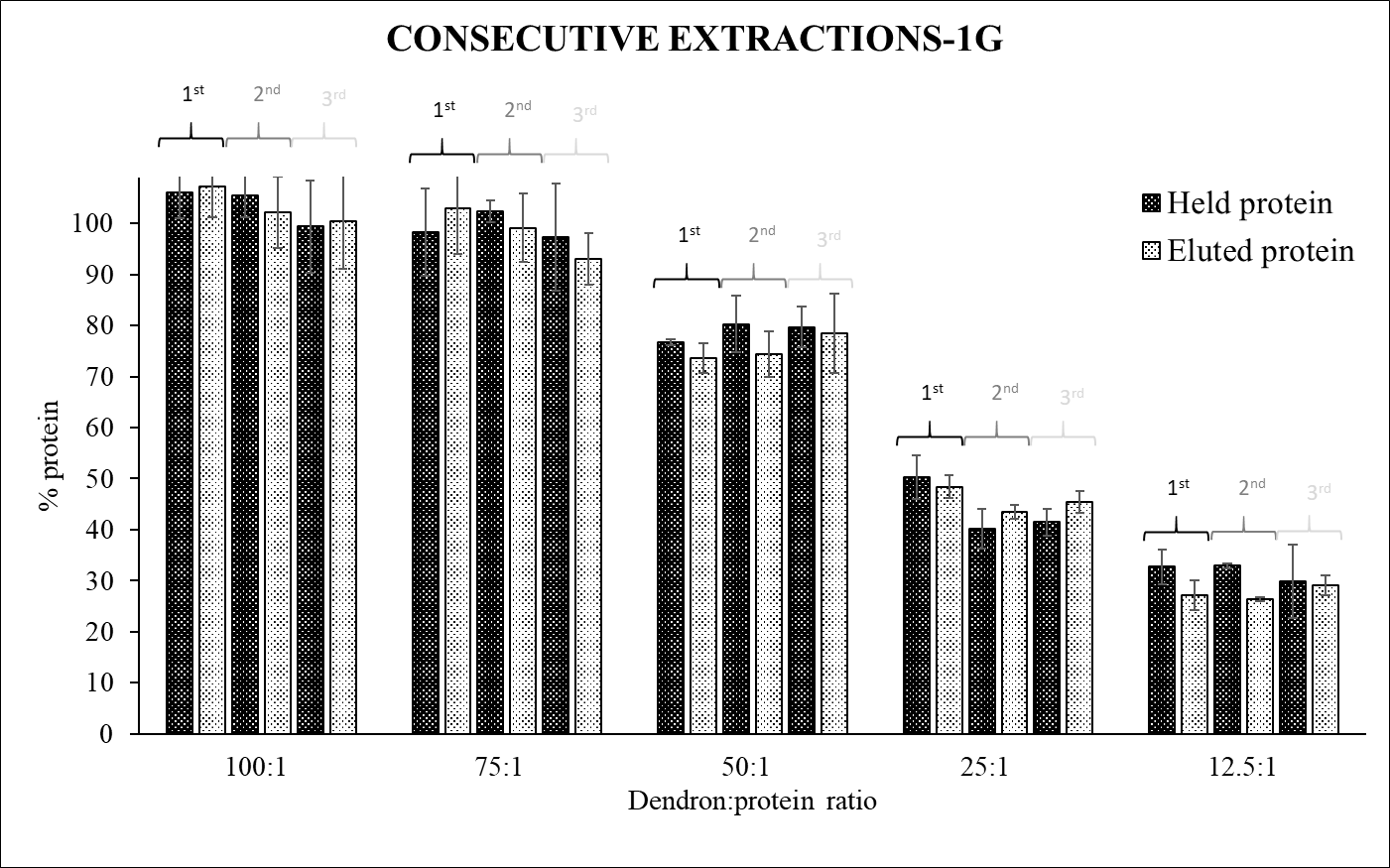


**Fig. S1.** Percentage of LYS retained and eluted from MNPs@G_1_(SCOOH)_2_ at basic pH in three consecutive experiments using different dendron:protein ratios.

**
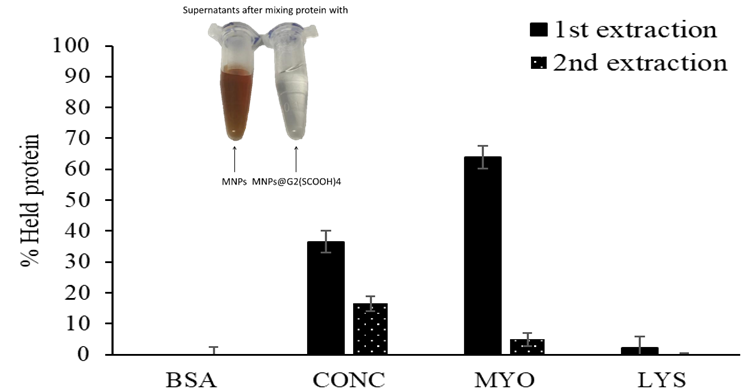
**

**Fig. S2**. Percentage of proteins (BSA, CONC, MYO, and LYS) retained on bared MNPs in two consecutive extractions at acid pH and detail of solutions remaining after extraction of proteins in the case of bared MNP and in the case of MNPs@G_2_(SCOOH)_4_.
